# Prevalence of frailty in Brazilian older adults: a systematic review and meta-analysis update

**DOI:** 10.1093/geroni/igaf122.2377

**Published:** 2025-12-31

**Authors:** Giselle Santos, Gabriela Cipolli, Ruth Melo

**Affiliations:** State University of Campinas, Campinas, Sao Paulo, Brazil; State University of Campinas, Campinas, Sao Paulo, Brazil; Universidade de São Paulo, São Paulo, Sao Paulo, Brazil

## Abstract

This systematic review investigates the prevalence of frailty syndrome in Brazil, considering different factors (context, diagnosis tool, and sex). Studies that included non-institutionalized older adults (60 years old) who were recruited from different contexts were considered. Studies must include a frailty assessment and a sample size of more than 281 participants. Indexed publications, dated from 2001 to October 2024, and retrieved from different databases were considered. COVID-19 related studies were excluded. Studies were independently selected by two reviewers and, in case of disagreements, a third reviewer was consulted. Prevalence data of included studies were meta-analyzed using the PERSyst-MA tool. Thirty-seven studies were included, totaling 82,955 participants. Studies were concentrated in the Southeast region (54%) and used the frailty phenotype (56.7%) as a diagnostic measure. The estimated prevalence of frailty was 21.6%, which was influenced by the assessment method and recruitment context. Frailty prevalence was lower when assessed with frailty phenotype criteria (16.7%) than Edmonton Frail Scale (27.7%). Higher frailty prevalence was observed in health services (32.3%) compared to primary care (23.2%) and community (16.1%). Frailty prevalence did not differ between men and women. These findings reveal significant variability in frailty prevalence among Brazilian older adults, influenced by the assessment tool and recruitment context. The present estimated prevalence underscores the substantial burden of this frailty in the Brazilian population. The results highlight the need for standardized frailty assessment methods and targeted public health strategies to address frailty across different settings.

